# Quality of life in older adults receiving hemodialysis: a qualitative study

**DOI:** 10.1007/s11136-019-02349-9

**Published:** 2019-11-05

**Authors:** Rasheeda K. Hall, Michael P. Cary, Tiffany R. Washington, Cathleen S. Colón-Emeric

**Affiliations:** 1 Renal Section, Durham Veterans Affairs Medical Center Healthcare System, Durham, NC USA; 2 Durham Veterans Affairs Healthcare System, Geriatric Research Education and Clinical Center, Durham VAMC, Durham, NC USA; 3 Division of Nephrology, Department of Medicine, Duke University, Box DUMC 2747, 2424 Erwin Road Suite 605, Durham, NC 27710 USA; 4 School of Nursing, Duke University, Durham, NC USA; 5 School of Social Work, University of Georgia, Athens, GA USA; 6 Division of Geriatric Medicine, Department of Medicine, Duke University, Durham, NC USA

**Keywords:** Aged, Patient-centered care, Social support, End-stage renal disease

## Abstract

**Purpose:**

Patient priorities for quality of life change with age. We conducted a qualitative study to identify quality of life themes of importance to older adults receiving dialysis and the extent to which these are represented in existing quality of life instruments.

**Methods:**

We conducted semi-structured interviews with 12 adults aged ≥ 75 years receiving hemodialysis to elicit participant perspectives on what matters most to them in life. We used framework analysis methodology to process interview transcripts (coding, charting, and mapping), identify major themes, and compare these themes by participant frailty status. We examined for representation of our study’s subthemes in the Kidney Disease Quality of Life (KDQOL-36) and the World Health Organization Quality of Life for Older Adults (WHOQOL-OLD) instruments.

**Results:**

Among the 12 participants, average age was 81 (4.2) years, 7 African-American, 6 women, and 6 met frailty criteria. We identified two major quality of life themes: (1) having physical well-being (subthemes: being able to do things independently, having symptom control, maintaining physical health, and being alive) and (2) having social support (subthemes: having practical social support, emotional social support, and socialization). Perspectives on the subthemes often varied by frailty status. For example, being alive meant surviving from day-to-day for frail participants, but included a desire for new life experiences for non-frail participants. The majority of the subthemes did not correspond with domains in the KDQOL-36 and WHOQOL-OLD instruments.

**Conclusion:**

Novel instruments are likely needed to elicit the dominant themes of having physical well-being and having social support identified by older adults receiving dialysis.

**Electronic supplementary material:**

The online version of this article (10.1007/s11136-019-02349-9) contains supplementary material, which is available to authorized users.

## Introduction

Most older adults receiving maintenance dialysis have limited life expectancy, multiple chronic conditions, and functional impairment [1–3]. Few interventions have been demonstrated to lengthen life or reduce debility in this population, making efforts to assess and optimize quality of life especially important [4].

The importance of quality of life assessment has been embraced by dialysis providers and regulatory bodies as well as patient organizations. For example, the Centers for Medicare and Medicaid Services (CMS) require annual assessment of quality of life, and the instrument commonly used is the Kidney Disease Quality of Life 36-item instrument (KDQOL-36), a shorter form of the original KDQOL. Although more than 25% of patients receiving dialysis is aged ≥ 75 years—the fastest growing group of new dialysis patients, only 10% of the patients who provided input on the KDQOL instrument were in this age group [5, 6]. Because older adults receiving dialysis differ from their younger counterparts in important ways, it is unclear whether this instrument reflects domains of quality of life that are important to these older patients. For example, older adults receiving dialysis tend to have more chronic conditions than their younger counterparts, a higher symptom burden, are less likely to be employed, have a shorter life expectancy, and have a higher prevalence of geriatric syndromes including frailty, functional impairment, and cognitive impairment [7, 8]. We have shown that these differences do not affect the psychometric properties of the KDQOL-36 in older adults [9]; however, they likely contribute to how older adults receiving dialysis view quality of life.

Studies of quality of life in non-dialysis populations have demonstrated that older adults prioritize different domains than younger adults do. In particular, older adults prioritize physical, psychological, social, and cognitive well-being, physical environment, spirituality, and end-of-life experiences [10–12]. As a result, quality of life instruments specific to older adults have been created, such as the WHOQOL-OLD [13]. Unlike older adults without renal failure, older adults receiving dialysis have additional unique life experiences that may impact their values, such as time required at a dialysis unit per week and post-dialysis fatigue. Therefore, it is not clear if quality of life instruments designed for older adults, such as the WHOQOL-OLD module, or the KDQOL-36, designed for younger patients on dialysis, encompass what matters most to those older adults receiving dialysis.

We conducted a qualitative study among older adults receiving maintenance hemodialysis to identify quality of life themes that matter most to older adults receiving dialysis and identify the extent to which existing quality of life instruments, specifically the KDQOL-36 and WHOQOL-OLD, overlap with those important themes. Our goal, consistent with the Institute for Healthcare Improvement’s 4 M Framework for creating age-friendly healthcare systems [14], was to ensure that we are able to capture what “Matters Most” to this clinically complex population.

## Methods

### Study design and population

We conducted semi-structured interviews with adults aged ≥ 75 years who received in-center hemodialysis in Durham, North Carolina. We included patients receiving hemodialysis for at least 3 months with at least two additional comorbid chronic conditions. We established a purposive sampling strategy to display the breadth of older adults receiving dialysis; therefore, we aimed to recruit at least two patients within each of the following categories: (1) frailty status (frail vs. not frail), (2) cognitive function (normal vs. abnormal cognitive screen), and (3) time on dialysis (greater than or less than 1 year). Because of the excess burden of end-stage renal disease in minority groups [15], we aimed to recruit at least 50% under-represented minority patients. Patients were ineligible if they had visual impairment, were unable to speak or understand English, had documented dementia, or were listed for kidney transplant. This study was approved by the Duke University Institutional Review Board. We report our study design and findings according to the consolidated criteria for reporting qualitative research (COREQ) checklist [16].

### Data collection procedures

The nephrologist principal investigator (PI) (RH) obtained informed consent for each participant and conducted all semi-structured interviews. Prior to study inception, the PI received qualitative research interview training and conducted mock interviews. The PI did not participate in routine patient care at the dialysis units; therefore, the PI did not have a prior or ongoing relationship with the study participants.

In the informed consent process, the PI informed participants that the purpose of the interview was to hear:” What matters most to you in life?” The interview guide was designed using a phenomenological perspective to yield a content analysis that explores patient experiences within the following domains: (1) journey to end-stage kidney disease, (2) life changes after dialysis initiation, and (3) important aspects of quality of life (Supplemental Material) [17]. The PI conducted each interview, lasting approximately 45 min to 1 h. The interviews were audiorecorded with the patients’ permission. Because patient interviews in the dialysis unit were attainable in other studies [18, 19], the PI allowed the participants to choose to have their interview during their dialysis session or at a location preferred by the participant. The PI maintained field notes and prepared memos after conducting each interview. Using a common approach for identifying saturation [20], the PI reviewed these memos for common broad themes (e.g., family support, independence). Using a spreadsheet, the PI compared those themes across transcripts to see if new themes emerged with each additional transcript. As is typical in other qualitative analyses [21, 22], after 12 interviews there were no new themes emerging from the interviews.

In addition to interviews, we obtained the following data from participants and/or their dialysis unit medical records: demographics (age, sex, and race), length of time on dialysis, type of residence, assistive device use, and comorbidities (were used to calculate a Charlson comorbidity index). We screened for cognitive impairment and frailty using the mini-cog and a simple frailty questionnaire (the FRAIL) [23], respectively.

### Data analysis

We used Atlas.ti qualitative data analysis software to code and organize interview transcripts for framework analysis [24]. Framework analysis is a methodology for content analysis that proceeds in five steps: (1) familiarization, (2) identifying a thematic framework, (3) indexing, (4) charting, and (5) mapping and interpretation. For familiarization, two investigators (RH and MC) read all transcripts. To identify a thematic framework, three investigators (RH, MC, and CCE) defined codes based on our interview guide questions, codes emerging from the data (i.e., the themes identified from memos), and our theoretical framework. Our theoretical framework was derived from a literature review of quality of life in older adults and includes the following quality of life domains: physical, psychological, social, and cognitive well-being, physical environment, spirituality, and experiences at the end-of-life experience [10–12]. With this defined codebook, we entered the indexing stage during which two investigators (RH and MC) coded each of the transcripts. During this stage, we discussed new codes that emerged from the data and reached an agreement before formally adding these to the codebook. For the charting stage, identical codes from each transcript were grouped together by code, and two investigators (RH and MC) prepared brief summaries of quotations assigned to the code. A separate investigator (CCE) reviewed all codes and quotations for completeness, accuracy, and authenticity. We created charts by arranging summaries for each code by participant. For the mapping and interpretation phase, we reviewed charts to identify similarities and differences across participants and identify subthemes. Because frailty is associated with perceived quality of life in older adults [25], we integrated participant frailty status with the charting, mapping, and interpretation stages to compare summaries and subthemes between frail and non-frail participants. Participants were not asked to compare quality of life instruments during the interviews so we compared themes identified in this study to domains (subscales) of the KDQOL-36 and the WHOQOL-OLD instruments. These commonly-used, validated instruments were selected because each were developed for unique populations; patients with kidney disease (KDQOL-36) and older adults (WHOQOL-OLD). As done in studies of quality of life in patients with chronic disease [26, 27], we mapped themes that emerged in this study to domains of those validated instruments to identify gaps in quality of life assessment for older adults receiving dialysis.

## Results

### Participant characteristics

Of 29 patients who received study recruitment letters, we conducted semi-structured interviews with 12 hemodialysis patients; 11 interviews were conducted in the dialysis clinic and 1 in a participant’s residence. Among the participants, there were 6 women, 7 African-Americans, and mean age was 81.0 (SD = 4.2) years. With respect to our purposive sampling strategy, six had a positive screen for frailty, of which one was a long-term care resident, three had an abnormal cognitive screen, and one had received dialysis for less than one year (mean time on dialysis was 5.4 (3.1) years) (Table 1).

**Table 1:** Study participant characteristics

Participant characteristic	Mean (SD) or *N* (%)
Age (years)	81 (4.2)
Female	6 (50%)
Race	
African-American	7 (58%)
White	4 (33%)
Asian	1 (8%)
Time on dialysis (years)	5.4 (3.1)
Charlson comorbidity index	8.3 (2.5)
Long-term care resident	1 (8.3%)
Assistive device use	7 (58%)
Abnormal cognitive screen (n = 11)	3 (27%)
Positive frailty screen	6 (50%)

### Summary of themes

**Fig. 1:**
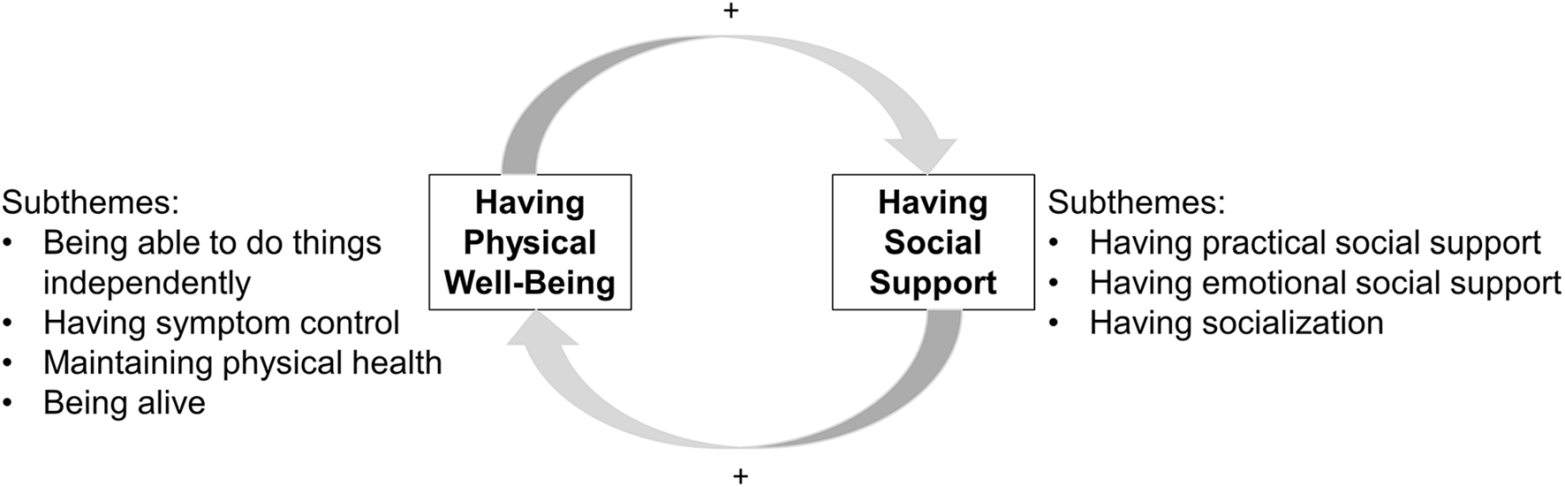
Schematic of quality of life themes and subthemes valued by older adults receiving hemodialysis

From the diverse experiences of the study participants, we identified two main aspects of quality of life that matter most to older adults receiving dialysis: having physical well-being and having social support. Having physical well-being subthemes included being able to do things independently, having symptom control, being alive, and maintaining current health status. Having social support subthemes included having practical social support, emotional social support, and socialization. These themes emerged among both frail and non-frail participants with an interplay relative to frailty status. Frail participants emphasized the value of social support, particularly practical social support (e.g., financial assistance, transportation, material aid) for quality of life and maintaining their health (Fig. 1). Non-frail participants appreciated how their physical well-being allowed them to engage with others for socialization and doing things they enjoy. Themes, subthemes, and key quotes are found in Table 2.

**Table 2:** Themes, subthemes and example quotes from study participantsn

Theme	Subtheme	Example quote
Having physical well-being	Being able to do things independently	“[To me, quality of life means] to be independent, which I am not now” (frail man, age 76) “I don’t like to have to have somebody to do every little thing for me.” (frail woman, age 88) “Yes sometimes when you’re sitting there and you can’t do what you want to do it makes you feel blue.” (non-frail woman, age 78) “Well when I get to the place where I can’t totally do anything for myself it wouldn’t be worth living” (non-frail woman, age 78)
	Having symptom control	“When I come home from dialysis I don’t [have] the energy that I used to have so I don’t do nearly as much as I used to.” (frail man, age 77) “Nobody knows what that cramp is like but it is death” (frail woman, age 88) “I felt wasted [after starting dialysis]. I wasn’t happy about it.” (non-frail man, age 83)
	Maintaining physical health	“I can’t think of anything that makes my life bad you know I’m healthy except for my kidney…” (frail woman, age 83) “I think that dialysis has helped my health” (non-frail man, age 77)
	Being alive	“Staying alive that’s the main thing” (frail woman, age 77) “So I know that I have to do this [dialysis] and I’m satisfied with that because I want to continue to live” (non-frail man, age 77)
Having social support	Having practical social support	“If they [staff] could get the van to come and go [to dialysis] on time.” (frail man, age 76) “[My daughters do] everything…I don’t have to do nothing.” (non-frail man, age 83)
	Having emotional social support	“It gives you a lift to know that people care and come in… and talk with you…if I didn’t have [support] from my family I couldn’t make it.” (frail woman, age 88) “I know I couldn’t make it without Him [God].“(non-frail woman, age 81) “somebody [dialysis staff] that you felt like they cared for you rather than just a job.” (non-frail woman, age 78)
	Having socialization	“…let me enjoy my family a little. Let me enjoy my precious grandsons and their family. I said because I don’t get to see my family. All I am doing is going to dialysis…” (frail woman, age 88) “Well I go out to lunch every Tuesday and Thursday. That’s a big thing in my life yeah it is” (non-frail man, age 83)

### Having physical well-being

#### Being able to do things independently

While level of independence varied among participants, almost all participants valued being able to do things without assistance from others. This ability was a source of “joy”. Many participants experienced decline in independence after starting dialysis. Having a new need for assistance introduced negative feelings including embarrassment, frustration, sadness, and worry about being a burden on family. To cope with loss of independence, some described that “it’s all in stride” and adjusted their routine to what they are physically able to do, recognizing it takes longer to do daily activities. Although most described acceptance of their current level of independence, some expressed worry about loss of additional abilities. For example, non-frail participants feared not being able to drive, while frail participants expressed concern about not being able to walk. Most described that loss of all ability to do things independently and needing total care would “make life not worth living”.

#### Having symptom control

Symptoms described by most participants were fatigue and pain. Specifically, most participants regardless of frailty status felt their quality of life was limited because they “don’t have the energy” that they had before starting dialysis. One frail participant noted, “dialysis helps one thing but it takes other things out of you”. Conversely, some non-frail participants deeply valued having energy to do things they desire to do when not at dialysis. Regarding pain, some participants reported pain associated with dialysis (e.g., needle insertion and cramps) and discomfort from prolonged sitting.

#### Maintaining physical health

Most participants noted that an important aspect of their quality of life is their current health status. Perceiving that their health was good “except for kidneys”, many value their health because they acknowledge it could worsen. This high value on physical health was a common motivation to attend dialysis regularly to maintain their health. Only non-frail participants conveyed that “dialysis has helped my health” and mentioned seeking help for other health concerns, such as sexual function. In contrast, frail participants tended to emphasize that they didn’t’ want their health to worsen and become more of a burden to their family.

#### Being alive

Related to maintaining their health, most participants viewed dialysis as “keeping them alive” and communicated that the most important thing to them was “staying alive”. This value, like maintaining health, is a primary motivation for attending dialysis regularly. Adjusting to dialysis often meant considering dialysis as a “job” as one participant stated, “it sets the schedule for our life”. Both frail and non-frail participants expressed they want to be alive for their family. However, frail participants perceived survival as a day-to-day goal, while non-frail participants expressed a longer time-perspective by conveying interest in having new life experiences.

### Having social support

#### Having practical social support

Irrespective of frailty status, most participants highly valued the support they received in activities of daily living, such as meal preparation, laundry, medication administration, financial management, and transportation. Many participants acknowledged importance of support from family and paid caregiver services, “I couldn’t make it without them”. The extent of support varied considerably. Those who had significant caregiving in the home noted they become “lazy” or less interested in doing things on their own. Some frail participants expressed the need for increased and/or improved IADL support. For example, frail participants who commonly rely on transportation services expressed frustration with waiting for or missing rides.

#### Having emotional social support

Most participants described the importance of having family, friends, and dialysis staff who provide emotional support. Emotional support was considered important for coping with health problems, particularly for having a family member present at clinic appointments or during a hospitalization. Some participants found emotional support through faith-based activities. Many participants appreciated dialysis staff who are compassionate and make them “feel like family”.

#### Having socialization

Both frail and non-frail participants commented on the value of having “fun” in social settings with family and friends. However, this socialization was reported to be limited by of the amount of time spent at dialysis. To address the time constraints, some participants have regularly planned gatherings with family and/or peers on non-dialysis days. Others commented on the value of connecting with other patients and staff in the dialysis unit.

### Comparing themes to validated quality of life instruments

KDQOL-36 domains were consistent with only two subthemes: being able to do things for oneself and symptom control. WHOQOL-OLD domains overlapped with only one subtheme of having physical well-being, and two of the three social support subthemes (Table 3). Notably absent in these validated instruments were domains pertaining to maintaining health, being alive, and having practical social support.

**Table 3:** Degree of overlap between key domains identified from interviews and domains of validated quality of life instruments

Key domains identified from interviews	KDQOL-36 domain	WHOQOL-OLD domain^a^
Having physical well-being		
Being able to do things for myself	SF-12 physical component score	Physical health; independence; autonomy; sensory abilities
Having symptom control	Symptoms of kidney disease	
Maintaining physical health		
Being alive		
Having social support		
Having practical social support		
Having emotional social support		Spirituality; social relations; intimacy
Having socialization		Intimacy; social participation

^a^Refers to domains of in the WHOQOL and the WHOQOL-OLD (an add-on module to the WHOQOL for administration to older adults)

## Discussion

In this qualitative study, we sought to identify the core values and essential aspects of quality of life among older adults receiving maintenance hemodialysis. Two dominant themes emerged from interviews with study participants related to quality of life: having physical well-being and having social support. These themes appeared to be relevant regardless of patients’ level of frailty, but how patients thought about physical well-being and social support appeared to be a dynamic property and varied according to their specific circumstances. We identified only limited overlap with existing quality of life instruments, KDQOL-36 and WHOQOL-OLD. Most subthemes identified were not consistently represented in those instruments, and there was no representation of subthemes related to maintaining physical health, being alive, and having practical social support. These findings highlight the need for a novel approach to quality of life assessment in older adults receiving maintenance hemodialysis that could be used to shape care, direct quality improvement efforts and assess the effect of interventions intended to improve quality of life.

This study identified quality of life priorities that are both similar and different from prior studies. Consistent with an international Delphi study of patients receiving dialysis [28], older adults interviewed for this study wanted to have more energy to do things and wanted to spend less time on dialysis. The value placed on staying alive and maintaining health among participants in this study is also consistent with the results of an ethnographic analysis that describes how patients viewed dialysis as a responsibility to continue despite worsening physical health [29]. On the other hand, unlike earlier work conducted among a cohort of adults with organ failure among whom end-of-life concerns figured prominently [10], most participants in this study valued “staying alive” and did not express concerns about how their life would end. While this may be explained by selection bias since all the prevalent dialysis patients in this study had already chosen dialysis over palliative care, this finding may also reflect that many study participants understood that dialysis extended their lives and if their health were to worsen then their responses may shift to discussions around end of life [30, 31]. Beyond this unexpected finding, our study also adds to the literature by emphasizing the high value of social support to older adults receiving dialysis. Social support, along with social participation and relationships, is considered part of social well-being, an important domain of quality of life for older adults [12]. When social well-being is limited, there is increased likelihood for declining health status [32, 33]. Therefore, close attention to social support is warranted as it is both valuable to the patient and to their health.

There was limited overlap between dominant themes identified in this study and existing instruments commonly used to assess quality of life in patients on dialysis and in older adults. While this finding highlights a limitation in our delivery of patient-centered care, it also introduces a potential opportunity to incorporate additional instruments for assessing those quality of life domains. Dialysis social workers who typically administer the KDQOL-36 perform additional routine assessments of physical limitations and psychosocial status. These assessments can uncover decrements in physical well-being and social support and be used to facilitate discussions at multidisciplinary dialysis unit rounds. A potential step forward could be adding those routine assessments to the KDQOL-36 as part of the CMS requirement for annual health-related quality of life assessment [34]. Another option is to incorporate validated items from the longer KDQOL-Short Form that describe how much time and support a patient receives from family and friends [35]. Beyond the KDQOL, the Medical Outcomes Study Social Support instrument may have utility as it includes 19 items that assess emotional, tangible support and social interactions, all themes identified in this study [36]. Social support could also be assessed through the Lubben Social Network Scale developed for older adults or social health items developed by the Patient-Reported Outcomes Measurement Information System (PROMIS) [37, 38]. Such PROMIS item banks are also available for assessing fatigue and physical function [39, 40]. While these alternative measures may be relatively quick to implement and enhance clinical practice in the short-term, the ideal solution is the development of a novel instrument specific to older adults receiving dialysis to ensure appropriateness of quality of life assessment.

Consistent with other literature on this relationship between frailty and quality of life, we found frail participants expressed a limited scope in desired activities and goals to achieve quality of life compared to non-frail participants [25]. As these perspectives can inform clinical decision-making, it is plausible that routine frailty assessment could improve the frequency and quality of goals of care discussions; these discussions can often be hard for patients to initiate [29]. Evidence suggests that frail older adults with good social support report better quality of life [41]. Novel social support interventions to target practical and emotional social support may help older adults receiving dialysis maintain or improve quality of life, especially for patients with declining health and/or limited family/friend interactions. For example, chronic disease self-management and geriatric assessment models of care that have been associated with improved self-efficacy and practical social support in older adults may yield improvements in quality of life in this population [42]. These hypotheses should be tested in future research.

Although we were able to obtain rich information on quality of life from older adults with considerable experience of life undergoing dialysis, our study has some limitations. Our study sample was diverse in frailty status and cognitive function. However, a more diverse sample will allow for further clarification of quality of life domains important to long-term care residents and older adults who recently started receiving dialysis. We recruited all participants from one geographic region and no participants identified as part of Hispanic ethnic group. To establish adequate content validity for a new quality of life instrument for older adults receiving dialysis, a larger more diverse sample would need to be engaged for concept elicitation and cognitive interviewing. Although most participants agreed to have an interview during a dialysis session, some responses may have been restrained because of privacy concerns and/or social desirability bias.

In summary, this qualitative study of quality of life in older adults receiving maintenance hemodialysis highlights the importance and intertwined nature of having physical well-being and having social support. These themes and their subthemes are not well represented by existing quality of life instruments. These findings suggest the need to develop new instruments and augment existing instruments to assess quality of life among older adults receiving maintenance dialysis. Broadly, these findings imply that older adults with chronic conditions have unique values in regards to quality of life which should be considered in both clinical practice and research.

## Electronic supplementary material

Below is the link to the electronic supplementary material.

Supplementary material 1 (DOCX 18 kb)
