# Supplementary material for: Quality of life in older adults receiving hemodialysis: a qualitative study
Source: Qual Life Res. 2019 Nov 5;29(3):655–63. doi: 10.1007/s11136-019-02349-9 (PMC7028790; doi:10.1007/s11136-019-02349-9)
Supplement: Supplementary file 1 — Supplementary material 1 (DOCX 18 kb) [file 11136_2019_2349_MOESM1_ESM.docx]

Quality of Life in Older Adults receiving Hemodialysis: A Qualitative Study

Quality of Life Research journal

Rasheeda K. Hall MD, MBA, MHS^1,2,3^ Michael P. Cary Jr, PhD, RN^4^ Tiffany R. Washington, PhD^5^ Cathleen S. Colón-Emeric, MD, MHS^2,6^

^1^Durham Veterans Affairs Medical Center Healthcare System, Renal Section, Durham, NC

^2^Durham Veterans Affairs Healthcare System, Geriatric Research Education and Clinical Center, Durham VAMC, Durham, NC

^3^Divison of Nephrology, Department of Medicine, Duke University, Durham, NC

^4^School of Nursing, Duke University, Durham, NC

^5^School of Social Work, University of Georgia, Athens, GA

^6^Divison of Geriatric Medicine, Department of Medicine, Duke University, Durham, NC

**Corresponding Author:** Rasheeda Hall MD, MBA, MHS, Box DUMC 2747**,** 2424 Erwin Road Suite 605, Durham, NC 27710, telephone: (919) 660-6861; fax: (919) 681-1143; email: [rasheeda.stephens@dm.duke.edu](mailto:rasheeda.stephens@dm.duke.edu)

**Table of Contents**

Participant Interview Guide 3

**Participant Interview Guide**

**Introduction**

You have agreed to an interview about life on dialysis. Through asking you questions, I hope to understand what is important to you in life. You do not have to answer every question and you can stop the interview at any point.

**Warm Up Questions**

1. How long have you been receiving dialysis?
2. How do you get to your dialysis sessions?
3. What do you typically do after dialysis and on your off days?

**Interview Questions**

1. Tell me the story of your kidney disease and how you ended up on dialysis.

- How did you learn you had a kidney problem
- How has kidney disease impacted the rest of your life?
- What kinds of treatments have you received for your kidney disease?
- How did you feel at that time?
- What was difficult about it?
- What did you do to cope?
- What did you do deal with challenges?
- Who if anyone was helpful?

1. How have things changed for you since you started dialysis?

- Any changes in your health?
- Any changes in your ability to do things?
- How did that make you feel?
- How have you adapted?
- What did you do to cope?
- Something good come from the change? Silver lining?

1. Tell me what has been most difficult for you about life on dialysis.

- Why has that been difficult
- What could be changed to make it better
- What do you think would be helpful in addressing this? Your needs?
- What would you most like help with,
- What could the staff here do to help? how could they be more helpful?

1. What could make life more difficult than it is now?
2. Tell me what has been most helpful for you about dialysis.
   - Why has that been helpful
   - Any benefits or things you look forward to when it comes to dialysis?
3. Tell me about any friend, family members or others who help you with dialysis and other aspects of your daily activities.

What kind of support do they provide?

How important is that support to you?

**Grand Tour Question:** What kinds of things are most important to you in life?

(what matters most to you in life)

Do you have any goals for the future?

Are there things that used to be important to you that don’t seem as important now?

- - Are there things that used to not be as important to you and that now are more important?
  - Could you imagine circumstance in which life wouldn’t be worth living anymore?
  - What would be most important to you if your health were to worsen?
  - What would be most important to you if you were not able to take care of yourself?

**Closing Question**

1. Is there anything I have missed or anything further that you would like to share with me?
